# Do Consumer’s Green Preference and the Reference Price Effect Improve Green Innovation? A Theoretical Model Using the Food Supply Chain as a Case

**DOI:** 10.3390/ijerph16245007

**Published:** 2019-12-09

**Authors:** Jianhong He, Yaling Lei, Xiao Fu

**Affiliations:** School of Economics and Management, Chongqing University of Posts and Telecommunications, Chongqing 400065, China; hejh@cqupt.edu.cn (J.H.); luohua_cqupt@hotmail.com (Y.L.)

**Keywords:** food supply chain, green innovation, green preference, reference price effect

## Abstract

Today, complex consumer purchase decisions affect company revolution, especially that dealing with environmental (green) innovation. Consumer preferences and pricing could interact in influencing green innovation. This situation is especially vital in industries that are characterized with many safety concerns that urge collaborative development between consumers and companies, such as the food industry. The aim of this paper is to explore the influence of the change in consumer preference characteristics on the green innovation efforts of the food supply chain, introducing the green preference of consumers and the reference price effect. A differential game model of green innovation in the food supply chain is constructed in this paper and solved. It was found that the change in consumer preference characteristics is an important factor to motivate supply chain members to make green innovation efforts. With the enhancement of consumer preference characteristics, the Pareto improvement effect of cost-sharing contracts on the profits of supply chain enterprises is clearer. Further, manufacturers can stimulate suppliers’ green innovation efforts through cost-sharing contracts. When the marginal profit ratio of food supply chain members reaches a certain threshold, the incentive effect of this cost-sharing contract is more significant, and it is more likely to realize the optimal profit of the food supply chain.

## 1. Introduction

Green innovation in the food supply chain is an innovative activity for manufacturers or suppliers in the food supply chain to reduce the energy consumption or emission of production processes by applying green product design, green materials and green production technologies [1,2]. It is an important measure to protect the ecological environment and accelerate the construction of a resource-saving and environment-friendly society in developing countries—it is taken as a necessary means to enhance competitiveness and gain competitive advantage [3] for enterprises, which attracts the extensive attention of researchers. Studies range from addressing the motivating factors of green innovation in the food supply chain [4,5,6] to the relative macro perspective of environmental regulation, technological change and market demand. Most studies are focused on relatively micro supply chain systems and include multi-agent decision-making mechanisms. Issues include whether cooperation and coordination among the main actors in the supply chain system can affect the results of green innovation [7] and how this can be achieved using methods such as cost-sharing contracts, wholesale price premium contracts and revenue-sharing contracts, etc. [8,9]. Generally speaking, most of the existing studies first address the supply side of green products and explore the driving factors and coordination methods for the green innovation efforts of enterprises [10,11].

However, the efficiency and sustainability of green innovation in the food supply chain are also affected by consumer preference characteristics and purchase decisions at the demand side. This follows the market diffusion and technological evolution of new technologies or products in other fields. It is especially true in the Chinese market, where the concept of green and sustainable development is gradually popularized. Consumer behaviors including risk aversion [12], low carbon preference [13], initial reference level [14] and information asymmetry [15] in green product purchase affect consumer purchase decision in terms of green innovation products and further affect the performance results of the green innovation efforts of food supply chain members.

Therefore, some researchers included consumer behavior preference in the green innovation decision-making process of the supply chain [16,17] and studied its effect on the green innovation efforts of supply chain enterprises [18,19]. It was found that green innovation is significantly related to consumer purchase intention and that consumer demand for green products in the food supply chain directly affects the green innovation efforts of enterprises. Consumers’ green preference can be used to implement green operation policy, and this can even help to gain additional market shares for companies with green strategies [20,21]. In this process, the reference price of traditional products not only affects consumer purchase decision, but also affects competitors’ strategic behavior [22,23], which may also interact with other characteristics of consumer preference. That is to say, when the product cost is pushed up by green innovation in the food supply chain and eventually shifts to the price of green products, consumers may have a very high reference price effect on green products due to the existence of the reference price effect. This effect can only be compromised by the intervention of other factors that consumers will prefer and even loyalty to products provided by green innovation in the supply chain [24,25].

With consideration of consumer preference characteristics at the demand side, it is worthwhile to re-discuss the pricing decision [26], product quality decision [27], equilibrium and competition [28,29], contract coordination [30,31] and other issues, because all of these factors may change in the green innovation of the food supply chain. This is especially true when new impacts on green innovation decision making in the food supply chain are created due to a change in consumer preference characteristics, and enterprises are more keen to cooperate with consumers to build and cultivate a green innovation system [32]. Today, consumer purchase decision has become increasingly complex. Consumer preferences and companies’ pricing could interact to influence green innovation. This situation is especially vital in industries that are characterized with many safety concerns that urge collaborative development between consumers and companies, such as the food industry. Based on this, the most basic elements of consumer preference characteristics, green preference and the reference price effect caused by the green preference, will be introduced in this paper. The independent and interactive effects of these two factors on the green innovation efforts of manufacturers and suppliers will be explored. We will also explore how manufacturers and suppliers can improve the profit level of a food supply chain system. Practical reference for managerial decisions will be discussed with the practices of cost-sharing contracts and coordination mechanisms on green innovation under the influence of the two aforementioned factors.

## 2. Problem Description and Model Hypothesis

### 2.1. Problem Description

In this paper, a two-level food supply chain consisting of upstream intermediate product suppliers (s) and downstream monopolized final product manufacturers (m) is constructed. Suppliers and manufacturers improve the green level of the whole food supply chain by arranging innovative activities such as using clean energy in production processes, introducing recyclable materials, developing new processes to reduce emissions and energy consumption, or conducting technology research and development and production. There are also other innovation products such as new products with low emission, recyclable products with other green properties, and ecological labels that are used to convey green information to consumers.

**Figure 1:**
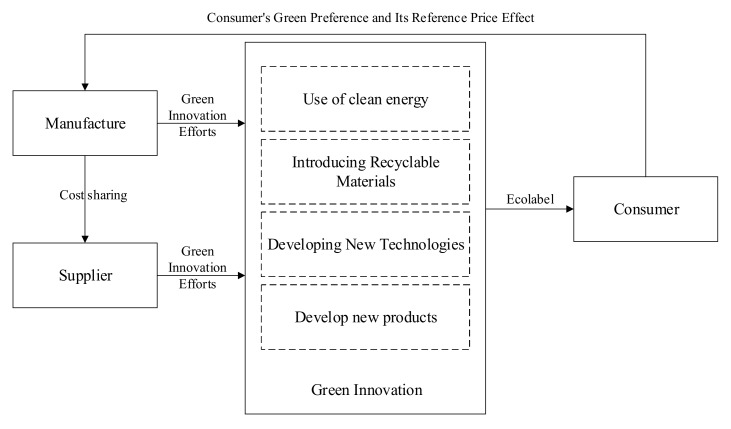
The green innovation process in the food supply chain.

Due to increased consumer awareness of environmental protection and sustainable development, products with green labels or ecological labels may be more popular in the market and may lead to higher sales revenues for manufacturers. In general supplier–manufacturer relationships, any incremental revenues mainly benefit the manufacturer, while the supplier can only obtain some indirect benefits, which limits the incentive of suppliers. However, the cost of green innovation is relatively high, and the process of innovation is uncertain. Manufacturers often hope that suppliers can make more efforts in terms of green innovation to effectively alleviate financial pressure and reduce the investment risk. In return, manufacturers are willing to provide certain subsidies for the green innovation efforts of suppliers, through cost-sharing contracts, so as to stimulate green innovation enthusiasm among suppliers and encourage suppliers to adopt the green strategy. At this stage, the green innovation process in the food supply chain is shown as the Figure 1.

### 2.2. Symbolic Explanation

This section refers to the definitions and specifications of the food supply chain field and defines and explains the common variables involved in the later supply chain game demonstration. These variables will be commonly used in the later demonstration process. The remaining parameters or variables in the paper will be further explained in the model assumptions.

$I_{i}(t)$ is the level of green innovation efforts of i at time t;

$C_{i}$ is the green innovation effort cost of i;

$k_{i}$ is the green innovation cost coefficient of i;

ϕ is the manufacturer’s share of the supplier’s green innovation cost;

$J_{i}$ is the net present value of the corporate profits of company i at time t;

$\Pi_{i}^{j}$ is the profit of company i at time j;

$\rho_{i}$ is the marginal profit per unit product of company i;

ρ is the discount rate.

Superscripts j={n,d,c} represent decentralized decision making under cost-free sharing, decentralized decision making under cost-sharing and decision-making scenarios under centralized decision making, respectively.

Subscripts i={m,s,sc} represent manufacturers, suppliers and food supply chain systems, respectively.

### 2.3. Model Hypothesis

(1) The cost of the green innovation efforts of food supply chain members is related to their level of green innovation effort. It increases with the increase in the level of green innovation effort, and the increase rate shows an upward trend. Considering the convexity of the green innovation effort cost, it is assumed that the green innovation cost of food supply chain members $I_{i}(t)$ is a convex function $C_{i}=\frac{1}{2}k_{i}I_{i}^{2}(t)$, i=m,s. Further, because green innovation belongs to a one-time scientific research input, it will not affect the fixed production cost of unit products.

(2) The reference price model is based on the combination of memory and stimulus, and it is assumed that the change in the reference price r(t) of a product obeys the following dynamic equation.

(1) $$\begin{array}{l} \overset{•}{r(t)}=\alpha [p-r(t)]+\beta [I_{m}(t)+I_{s}(t)]\\ r(0)=r_{0} \end{array}$$

r(t) is the reference price of products at time t;

$\overset{•}{r(t)}=\frac{dr(t)}{dt}$ is the change rate of the reference price r(t) at time t;

$r_{0}$ is the initial reference price of the product i;

α(α>0) is the “memory parameter” of consumers; the larger the reference price is, the clearer the short-term memory of consumers in terms of the previous market price and related purchase experience—α=0 means that the consumer has no memory of the previous market price of the product;

β(β>0) is the impact factor of green innovation efforts on the reference price.

(3) On the basis of the classical demand function a−bp, two factors (reference price effect and consumers’ green preference) are introduced to express consumer demand function for green products in green supply chain t, and the formula is as follow.

(2) $$Q(t)=a-bp+\delta [r(t)-p]+\eta [I_{m}(t)+I_{s}(t)]$$

The above shows that consumer demand for green products is affected by the sales price of green products, the level of greenness and the reference price of green products. Among them, a(a>0) is the potential market demand of green products, b(b∈(0,1)) is the price elasticity coefficient of demand, and Q.δ(δ>0) is the reference price coefficient, indicating the sensitivity of consumers to the actual price and the reference price, which reflects the reference price effect. In special situations, δ=0 means that there is no reference price effect. In addition, η(η∈[0,1]) is the consumers’ green level sensitivity coefficient, which shows the preference level of consumers’ green choice. The greater η is, the greater the consumer’s preference for green products is.

## 3. Model Construction and Solution

In this paper, the green innovation efforts of a food supply chain system consisting of a manufacturer and a supplier are transformed into system optimal control problems. With the help of game theory and Hamilton–Jacobi–Bellman (HJB) equations, the problems are determined from the dimensions of centralized decision making, decentralized decision making, and cost-sharing scenarios. Analytic method selection is based on the following considerations. First is universality. Game theory is a mathematical theory and method for studying the phenomena of struggle or competition. It is widely used in the research of supply chain problems. Second is exploratory. This article analyzes the green innovation efforts of the food supply chain from the perspective of system optimal control. This is an effective attempt to combine engineering theory with ecological practice. Third is effectiveness. Starting from a long-term effective mechanism, the scenario of maximizing the profit of supply chain members and the entire system is discussed separately, which is conducive to proposing an executable supplier cost subsidy program.

Based on the description of the problem in the previous section, this section further provides an analysis about whether the cost-sharing contract can make the optimal decision of the manufacturer and supplier reach the optimal level in centralized decision making. If the optimal level in centralized decision making cannot be reached, then the analysis of the cost-sharing contract is required to test if it can make the optimal decision of the manufacturer and supplier reach the degree of Pareto improvement, so as to provide the decision-making basis of green innovation efforts in the food supply chain.

### 3.1. Centralized Decision Making

With the centralized decision-making method, manufacturers and suppliers in the supply chain have reached a binding cooperation agreement on green innovation, which provides a decision-making base for both partners to maximize the profits of the whole supply chain system. So the manufacturers and suppliers are considered as a whole to determine their optimal level of green innovation efforts $I_{m}(t)$ and $I_{s}(t)$. Therefore, centralized decision making should be analyzed in the first place, and so the decision-making problem of the whole supply chain is as follows:

(3) $$J_{sc}^{c}(r,t)=\underset{I_{m}(t),I_{s}(t)}{\max}\int_{0}^{\infty }e^{-\rho t}\{(\rho_{m}+\rho_{s})Q-C_{m}(I_{m}(t))-C_{s}(I_{s}(t))\}dt$$

In order to obtain the feedback equilibrium strategy of supply chain members, the Hamilton–Jacobi–Bellman equation (hereinafter referred to as HJB equation) is used to solve the problem.

Referring to the solution of optimal control, it can be seen from Formula (2) that the optimal profit value function of the supply chain system at time *t* is

(4) $$J_{sc}^{c*}(r,t)=\underset{I_{m},I_{s}}{\max}\int_{t}^{\infty }e^{-\rho s}\{(\rho_{m}+\rho_{s})Q-C_{m}(I_{m})-C_{s}(I_{s})\}ds$$

Thus, the optimal profit value function of the supply chain system at time *t* is transformed into

(5) $$J_{sc}^{c*}(r,t)=e^{-\rho t}V_{sc}^{c}(r)$$

At this point, the optimal control problem of the supply chain system satisfies the following HJB equation.

(6) $$\rho V_{sc}^{c}(r)=\underset{I_{m},I_{s}}{\max}\{(\rho_{m}+\rho_{s})Q-C_{m}(I_{m})-C_{s}(I_{s})+V_{sc}^{c^{’}}(r)\overset{•}{r}\}$$

By solving the optimal control problem, the optimal level of green innovation efforts of manufacturers and suppliers can be obtained as follows:

(7) $$I_{m}^{c*}=\frac{\left(\rho_{m}+\rho_{s})(\rho \eta +\alpha \eta +\beta \delta \right)}{k_{m}(\rho +\alpha )},\;I_{s}^{c*}=\frac{\left(\rho_{m}+\rho_{s})(\rho \eta +\alpha \eta +\beta \delta \right)}{k_{s}(\rho +\alpha )}$$

If Formula (5) is introduced into the equation of state (1), according to the boundary conditions of the equation of state, the response function of the consumer’s reference price can be obtained as follows.

(8) $$\begin{array}{l} r^{c*}(t)=[r_{0}-p-\frac{\beta (k_{m}+k_{s})(\rho_{m}+\rho_{s})(\eta \rho +\eta \alpha +\beta \delta )}{\alpha k_{m}k_{s}(\rho +\alpha )}]e^{-\alpha t}+p\\ +\frac{\beta (k_{m}+k_{s})(\rho_{m}+\rho_{s})(\eta \rho +\eta \alpha +\beta \delta )}{\alpha k_{m}k_{s}(\rho +\alpha )} \end{array}$$

The optimal profit value function of the supply chain system can be obtained as follows.

(9) $$J_{sc}^{c*}(r,t)=e^{-\rho t}(a_{1}^{c*}r+b_{1}^{c*})$$

and $a_{1}^{c*}=\frac{(\rho_{m}+\rho_{s})\delta }{\rho +\alpha }$, $\begin{array}{l} b_{1}^{c*}=\frac{\left(\rho_{m}+\rho_{s}\right)}{\rho }\{a-bp-\delta p+\frac{\eta (k_{m}+k_{s})[(\rho_{m}+\rho_{s})\eta +\beta a_{1}]}{k_{m}k_{s}}\}\\ -\frac{(k_{m}+k_{s}){\left[(\rho_{m}+\rho_{s})\eta +\beta a_{1}\right]}^{2}}{2\rho k_{m}k_{s}}+\frac{a_{1}}{\rho }\{\alpha p+\frac{\beta (k_{m}+k_{s})[(\rho_{m}+\rho_{s})\eta +\beta a_{1}]}{k_{m}k_{s}}\} \end{array}$.

### 3.2. Decentralized Decision Making without Cost Sharing

The analysis of cost-free decentralized decision making can be used to consider the participation constraints of manufacturers and suppliers in contract design. It also provides a reference basis for the effect of contract coordination. If the optimal level of centralized decision making is the upper limit of contract coordination, then the optimal level of decentralized decision making without cost sharing is the lower limit of profits of all parties in contract coordination. With decentralized decision making without cost sharing, manufacturers and suppliers can make decisions independently to maximize their respective profits.

The decision-making problems of manufacturers and suppliers are as follows:

(10) $$\underset{I_{m}}{\max}J_{m}^{n}=\int_{0}^{\infty }e^{-\rho t}\{\rho_{m}Q-C_{m}(I_{m})\}dt$$

(11) $$\underset{I_{s}}{\max}J_{s}^{n}=\int_{0}^{+\infty }e^{-\rho t}\{\rho_{s}Q-C_{s}(I_{s})\}dt$$

In order to obtain the feedback equilibrium strategy of each member of the supply chain, the HJB equation is used to solve the problem. The optimal control problem of the supplier should be solved first. The optimal profit value function of the supplier at time *t* is determined by Formula (9). At this point, the supplier optimal control problem satisfies the following HJB equation

(12) $$\begin{array}{l} \rho V_{s}^{n}(r)=\underset{I_{s}}{\max}\{\rho_{s}[a-bp+\delta (r-p)+\eta (I_{m}+I_{s})]-\frac{1}{2}k_{s}I_{s}^{2}\\ +V_{s}^{n^{’}}(r)[\alpha (p-r)+\beta (I_{m}+I_{s})]\} \end{array}$$

At this point, the manufacturer’s optimal control problem satisfies the following HJB equation

(13) $$\rho V_{m}^{n}(r)=\underset{I_{m}}{\max}\{\rho_{m}Q-C_{m}+V_{m}^{n^{’}}(r)\overset{•}{r}\}$$

By solving the optimal control problem, the optimal level of green innovation efforts of manufacturers and suppliers can be obtained as follows:

(14) $$I_{m}^{n*}=\frac{\rho_{m}(\rho \eta +\alpha \eta +\beta \delta )}{k_{m}(\rho +\alpha )},\;I_{s}^{n*}=\frac{\rho_{s}(\rho \eta +\alpha \eta +\beta \delta )}{k_{s}(\rho +\alpha )}$$

If Equation (12) is introduced into the equation of state (1), the response function of the consumer’s reference price can be obtained as follows according to the boundary conditions of the equation of state.

(15) $$r^{c*}(t)=[r_{0}-p-\frac{\beta (k_{m}\rho_{s}+k_{s}\rho_{m})(\eta \rho +\eta \alpha +\beta \delta )}{\alpha k_{m}k_{s}(\rho +\alpha )}]e^{-\alpha t}+p+\frac{\beta (k_{m}\rho_{s}+k_{s}\rho_{m})(\eta \rho +\eta \alpha +\beta \delta )}{\alpha k_{m}k_{s}(\rho +\alpha )}$$

Then the optimal profit function of manufacturer and supplier can be obtained as follows:

(16) $$\left\{\begin{array}{l} J_{m}^{n*}(r,t)=e^{-\rho t}(a_{1}^{n*}r+b_{1}^{n*})\\ J_{s}^{n*}(r,t)=e^{-\rho t}(a_{2}^{n*}r+b_{2}^{n*}) \end{array}\right.\;\{\begin{array}{l} a_{1}^{n*}=\frac{\rho_{m}\delta }{\rho +\alpha }\\ a_{2}^{n*}=\frac{\rho_{s}\delta }{\rho +\alpha }\\ b_{1}^{n*}=\frac{\rho_{m}}{\rho }[a-bp-\delta p+\eta (\frac{\rho_{m}\eta +\beta a_{1}^{n}}{k_{m}}+\frac{\rho_{s}\eta +\beta a_{2}^{n}}{k_{s}})]\\ -\frac{{\left(\rho_{m}\eta +\beta a_{1}^{n}\right)}^{2}}{2\rho k_{m}}+\frac{a_{1}^{n}}{\rho }[\alpha p+\beta (\frac{\rho_{m}\eta +\beta a_{1}^{n}}{k_{m}}+\frac{\rho_{s}\eta +\beta a_{2}^{n}}{k_{s}})]\\ b_{2}^{n*}=\frac{\rho_{s}}{\rho }[a-bp-\delta p+\eta (\frac{\rho_{m}\eta +\beta a_{1}^{n}}{k_{m}}+\frac{\rho_{s}\eta +\beta a_{2}^{n}}{k_{s}})]\\ -\frac{{\left(\rho_{s}\eta +\beta a_{2}^{n}\right)}^{2}}{2\rho k_{s}}+\frac{a_{2}^{n}}{\rho }[\alpha p+\beta (\frac{\rho_{m}\eta +\beta a_{1}^{n}}{k_{m}}+\frac{\rho_{s}\eta +\beta a_{2}^{n}}{k_{s}})] \end{array}$$

### 3.3. Decentralized Decision Making under Cost Sharing

Assumption: In decentralized decision making of the cost sharing, the manufacturer plays a leading role in the green innovation of the supply chain, and the supplier plays a follower role accordingly. In order to motivate the supplier to carry out green innovation, the manufacturer provides a certain proportion of subsidies to the supplier. Then, from a long-term and dynamic point of view, the decision making between manufacturers and suppliers on the level of green innovation effort constitutes a Stackelberg differential game model between upstream and downstream.

Manufacturers and suppliers make decisions independently to maximize their respective profits. In the first stage, the manufacturer decides its own level of green innovation effort $I_{m}(t)$ and the proportion ϕ(t) of green innovation cost that the manufacturer promised to supplier. In the second stage, according to the given sum $I_{m}(t)$ and ϕ(t), the supplier decides its own level of green innovation effort $I_{s}(t)$. Firstly, the manufacturer’s decision-making problem could be formulated as follows:

(17) $$\underset{I_{m},\phi }{\max}J_{m}^{d}=\int_{0}^{\infty }e^{-\rho t}\{\rho_{m}Q-C_{m}(I_{m})-\phi C_{s}(I_{s})\}dt$$

Given $I_{m}$ and ϕ, the supplier’s decision problem is

(18) $$\underset{I_{s}}{\max}J_{s}^{d}=\int_{0}^{\infty }e^{-\rho t}\{\rho_{s}Q-(1-\phi )C_{s}(I_{s})\}dt$$

At this point, the supplier optimal control problem satisfies the following HJB equation

(19) $$\rho V_{s}^{d}(r)=\underset{I_{s}}{\max}\{\rho_{s}[a-bp+\delta (r-p)+\eta (I_{m}+I_{s})]-\frac{1}{2}(1-\phi )k_{s}I_{s}^{2}+V_{s}^{d^{’}}(r)[\alpha (p-r)+\beta (I_{m}+I_{s})]\}$$

At this point, the manufacturer’s optimal control problem satisfies the following HJB equation

(20) $$\rho V_{m}^{d}(r)=\underset{I_{m},\phi }{\max}\{\rho_{m}Q-C_{m}-\phi C_{s}+V_{m}^{n^{’}}(r)\overset{•}{r}\}$$

By solving the optimal control problem, the equilibrium solution of manufacturer and supplier can be obtained as follows.

(21) $$I_{m}^{d*}=\frac{\rho_{m}(\eta \rho +\eta \alpha +\delta \beta )}{k_{m}(\rho +\alpha )},\;\phi^{d*}=\frac{2\rho_{m}-\rho_{s}}{2\rho_{m}+\rho_{s}},\;I_{s}^{d*}=\frac{\left(2\rho_{m}+\rho_{s})(\eta \rho +\eta \alpha +\delta \beta \right)}{2k_{s}(\rho +\alpha )}$$

Equation (19) is introduced into the equation of state (1). According to the boundary conditions of the equation of state, the response function of the consumer’s reference price can be obtained as follows.

(22) $$r^{d*}(t)=[r_{0}-p-\frac{\beta (k_{m}\rho_{s}+2k_{s}\rho_{m}+2k_{m}\rho_{m})(\eta \rho +\eta \alpha +\beta \delta )}{2\alpha k_{m}k_{s}(\rho +\alpha )}]e^{-\alpha t}+p+\frac{\beta (k_{m}\rho_{s}+2k_{s}\rho_{m}+2k_{m}\rho_{m})(\eta \rho +\eta \alpha +\beta \delta )}{2\alpha k_{m}k_{s}(\rho +\alpha )}$$

The optimal profit function of manufacturer and supplier can be obtained as follows.

(23) $$\left\{\begin{array}{l} J_{m}^{d*}(r,t)=e^{-\rho t}(a_{1}^{d*}r+b_{1}^{d*})\\ J_{s}^{d*}(r,t)=e^{-\rho t}(a_{2}^{d*}r+b_{2}^{d*}) \end{array}\right.\;\left\{\begin{array}{c} a_{1}^{d*}=\frac{\rho_{m}\delta }{\rho +\alpha }\\ a_{2}^{d*}=\frac{\rho_{s}\delta }{\rho +\alpha }\\ b_{1}^{d*}=\frac{\rho_{m}}{\rho }\left[a-bp-\delta p+\eta \left(\frac{\rho_{m}\eta +\beta a_{1}^{d}}{k_{m}}+\frac{2\rho_{m}\eta +\rho_{s}\eta +2\beta a_{1}^{d}+\beta a_{2}^{d}}{2k_{s}}\right)\right]-\frac{{\left(\rho_{m}\eta +\beta a_{1}^{d}\right)}^{2}}{2\rho k_{m}}\\ -\frac{{\left(2\rho_{m}\eta +2\beta a_{1}^{d}\right)}^{2}-{\left(\rho_{s}\eta +\beta a_{2}^{d}\right)}^{2}}{8\rho k_{s}}+\frac{a_{1}^{d}}{\rho }\left[\alpha p+\beta \left(\frac{\rho_{m}\eta +\beta a_{1}^{d}}{k_{m}}+\frac{2\rho_{m}\eta +\rho_{s}\eta +2\beta a_{1}^{d}+\beta a_{2}^{d}}{2k_{s}}\right)\right]\\ b_{2}^{d*}=\frac{\rho_{s}}{\rho }\left[a-bp-\delta p+\eta \left(\frac{\rho_{m}\eta +\beta a_{1}^{d}}{k_{m}}+\frac{2\rho_{m}\eta +\rho_{s}\eta +2\beta a_{1}^{d}+\beta a_{2}^{d}}{2k_{s}}\right)\right]-\frac{{\left(2\rho_{m}\eta +\rho_{s}\eta +2\beta a_{1}^{d}+\beta a_{2}^{d}\right)}^{2}}{8\rho k_{s}}\\ +\frac{{\left(2\rho_{m}\eta +2\beta a_{1}^{d}\right)}^{2}-{\left(\rho_{s}\eta +\beta a_{2}^{d}\right)}^{2}}{8\rho k_{s}}+\frac{a_{2}^{d}}{\rho }\left[\alpha p+\beta \left(\frac{\rho_{m}\eta +\beta a_{1}^{d}}{k_{m}}+\frac{2\rho_{m}\eta +\rho_{s}\eta +2\beta a_{1}^{d}+\beta a_{2}^{d}}{2k_{s}}\right)\right] \end{array}\right.$$

**Proposition** **1.**  *When the supplier’s marginal profit is less than twice the manufacturer’s marginal profit, compared with the decentralized decision making without cost sharing, the optimal level of the green innovation effort of manufacturers remains unchanged under the decentralized decision making with cost sharing, and the optimal level of the green innovation effort of suppliers is improved. When the supplier’s marginal profit is greater than twice the manufacturer’s marginal profit, the optimal level of the green innovation efforts of manufacturers remains unchanged with the decentralized decision making with cost sharing, while the optimal level of the green innovation efforts of suppliers decreases.*

**Proof** **of** **Proposition** **1.**  (24) $I_{m}^{d*}-I_{m}^{n*}=\frac{\rho_{m}(\eta \rho +\eta \alpha +\delta \beta )}{k_{m}(\rho +\alpha )}-\frac{\rho_{m}(\eta \rho +\eta \alpha +\delta \beta )}{k_{m}(\rho +\alpha )}=0\;I_{s}^{d*}-I_{s}^{n*}=\frac{\left(2\rho_{m}+\rho_{s})(\eta \rho +\eta \alpha +\delta \beta \right)}{2k_{s}(\rho +\alpha )}-\frac{\rho_{s}(\rho \eta +\alpha \eta +\beta \delta )}{k_{s}(\rho +\alpha )}=\frac{\left(2\rho_{m}-\rho_{s})(\rho \eta +\alpha \eta +\beta \delta \right)}{2k_{s}(\rho +\alpha )}$

When $\rho_{s}>2\rho_{m}$, $I_{s}^{d*}<I_{s}^{n*}$; when $\rho_{s}<2\rho_{m}$, $I_{s}^{n*}<I_{s}^{d*}$.

Proposition 1 show that the cost-sharing contract has no effect on the manufacturer’s own optimal level of green innovation effort, but it will affect the optimal level of the green innovation effort of suppliers. Under the same conditions, the lower the cost of green innovation efforts, the more willing suppliers are to improve their green innovation efforts in order to seek a more long-term and stable cooperative relationship with manufacturers. Suppliers’ enthusiasm for green innovation efforts is closely related to their marginal profits and suppliers’ marginal profits. When their marginal profits are less than the manufacturers’ marginal profits, suppliers are more willing to follow manufacturers in green innovation efforts. Although the marginal profit of supplier is less than twice that of the manufacturer, this is a necessary condition for the establishment of a cost-sharing contract.□

**Corollary** **1.**  *The marginal profit ratio of supplier and manufacturer has a threshold. Within the threshold range, a cost-sharing contract has an obvious incentive effect on the green innovation efforts of suppliers. Once beyond the threshold range, the green innovation efforts of suppliers may depend more on the influence of other factors, and the incentive effect of a cost-sharing contract on the green innovation efforts of suppliers is greatly reduced.*

**Proposition** **2.**  *The optimal level of the green innovation efforts of manufacturers under centralized decision making is higher than that of manufacturers under cost sharing. The optimal level of the green innovation efforts of suppliers under centralized decision making is higher than that of suppliers under cost sharing.*

**Proof** **of** **Proposition** **2.**  (25) $I_{m}^{c*}-I_{m}^{d*}=\frac{\left(\rho_{m}+\rho_{s})(\rho \eta +\alpha \eta +\beta \delta \right)}{k_{m}(\rho +\alpha )}-\frac{\rho_{m}(\eta \rho +\eta \alpha +\delta \beta )}{k_{m}(\rho +\alpha )}=\frac{\rho_{s}(\eta \rho +\eta \alpha +\delta \beta )}{k_{m}(\rho +\alpha )}>0\;I_{s}^{c*}-I_{s}^{d*}=\frac{\left(\rho_{m}+\rho_{s})(\rho \eta +\alpha \eta +\beta \delta \right)}{k_{s}(\rho +\alpha )}-\frac{\left(2\rho_{m}+\rho_{s})(\eta \rho +\eta \alpha +\delta \beta \right)}{2k_{s}(\rho +\alpha )}=\frac{\rho_{s}(\eta \rho +\eta \alpha +\delta \beta )}{2k_{s}(\rho +\alpha )}>0$

Proposition 2 shows that the optimal level of the green innovation efforts of manufacturers and suppliers under centralized decision making is the highest and it is also the upper limit for the whole supply chain system to carry out green innovation efforts. Although a cost-sharing contract can improve the optimal level of green innovation efforts of manufacturers and suppliers to a certain extent, it cannot achieve the Pareto optimum. For manufacturers, in order to improve the incentive effect of cost-sharing contracts, it is necessary to reduce the cost of the green innovation efforts of manufacturers and increase the marginal profits of suppliers. For suppliers, in order to improve the incentive effect of cost-sharing contracts, it is necessary to reduce the cost of the green innovation efforts of suppliers and increase suppliers’ marginal profits.□

**Proposition** **3.**  *Under decentralized decision making with cost sharing, the cost sharing ratio of manufacturer to supplier is positively correlated with the marginal profit of the manufacturer and negatively correlated with the marginal profit of the supplier.*

**Proof** **of** **Proposition** **3.**  (26) $\phi =\frac{2\rho_{m}-\rho_{s}}{2\rho_{m}+\rho_{s}},\;\frac{\partial \phi }{\partial \rho_{m}}=\frac{4\rho_{s}}{{\left(2\rho_{m}+\rho_{s}\right)}^{2}}>0,\;\frac{\partial \phi }{\partial \rho_{s}}=-\frac{4\rho_{m}}{{\left(2\rho_{m}+\rho_{s}\right)}^{2}}<0,\;\frac{\partial^{2}\phi }{\partial \rho_{m}^{2}}=-\frac{16\rho_{s}}{{\left(2\rho_{m}+\rho_{s}\right)}^{3}}<0,\;\frac{\partial^{2}\phi }{\partial \rho_{s}^{2}}=\frac{8\rho_{m}}{{\left(2\rho_{m}+\rho_{s}\right)}^{3}}>0.$

Proposition 3 shows that the cost-sharing ratio of the manufacturer to the supplier increases with the increase in the marginal profit of the manufacturer and decreases with the increase in the marginal profit of the supplier. In the whole life cycle of green innovation efforts, the cost-sharing ratio of manufacturers to suppliers is a dynamic changing process. Manufacturers will constantly adjust the cost-sharing ratio of suppliers according to the actual situation, so as to ensure the maximization of both sides’ green innovation benefits and promote improvement in the green innovation efficiency of the supply chain system. Therefore, in order to improve the cost-sharing ratio between manufacturers and suppliers, it is necessary to increase the marginal profit of manufacturers while reducing the marginal profit of suppliers. The increase in cost-sharing ratio can increase the enthusiasm of suppliers for green innovation efforts, but it may not necessarily improve the optimal level of the green innovation efforts of manufacturers.□

## 4. Data Simulation

### 4.1. Simulation Method and Parameter Setting

In order to better illustrate the application of the model, the validity of the above three green innovation strategies will be tested by the numerical simulation of Mathematica, and the impact of consumers’ green preference and the reference price effect on the profit of the supply chain system will be discussed.

Mathematica was selected based on the following reasons: (1) the tool can perform complex mathematical operation analysis and directly provide graphical analysis results, which is the ideal result for the simulation analysis of this article; (2) the method is widely used in supply chain studies. The numerical simulation of the domain model has been highly recognized by researchers in the field. Specifically, this paper uses the Mathematica system to numerically simulate the game model of the food supply chain system under the three decision situations constructed above in order to obtain the changes in profits caused by the green innovation efforts of food supply chain members (within a certain period of time). The simulation results described how consumers’ green preferences and reference price effects interact with the green innovation efforts of the food supply chain. Moreover, the feasibility of the effectiveness mechanism issue provides factual arguments.

In the simulation analysis, the initial parameters of the model are derived from parameters set in similar literature as well as data obtained through a small experiment designed by the authors. In the experiment, there were 60 participants aged between 18 and 50 years old—40% were male and 60% were female. The consumer goods in the experiment were organic milk and ordinary milk produced by the same manufacturer. In order to understand the participants’ green preferences for milk products and the corresponding price sensitivity, we designed a brief questionnaire for participants to complete. Finally, we adopted the mean answers as parameters for the simulation model.

The following assumptions are made α=2, β=0.5, ρ=0.1, a=20, b=1, p=20, $\rho_{m}=10$, $\rho_{s}=8$, $k_{m}=5$, $k_{s}=3$, $r_{0}=7$, δ=1, η=0.5 using parameters from green innovation-related literature [33,34] and the results of the experiment in this paper. α=2 shows that consumers have a clear memory of the previous market and purchase experience of ordinary products. Therefore, the reference price $r_{0}=7$ of green products in their minds is lower than the actual price p=20 of products. On a green sensitivity coefficient level of η=0.5, the green degree of products brought by the green innovation efforts and related information transmitted by ecological labels prompt consumers to change their reference prices. That is, the influence factor of green product innovation on the reference prices is β=0.5. Because consumers are sensitive to the difference between the actual price of the product and the reference price, which makes δ=1, consumers will make a decision to buy the green products when the reference price of the consumer is infinitely close to the actual price of the green products.

### 4.2. Simulation Results

To obtain simulative results, we assigned values for variables $a,b,r,p,r_{m},r_{s},k_{m},k_{s},r_{0},\delta ,\eta ,\beta$. Further, we applied the “Simplify” function to categorize three decisional conditions for suppliers, manufacturers, and the supply chain as a whole. Next, the solutions for higher-level, complex parameters have been estimated. Then, we used the “PLOT” function to draw the simulation results visually. These procedures have been conducted recursively 10 times for a better simulation result.

**Figure 2:**
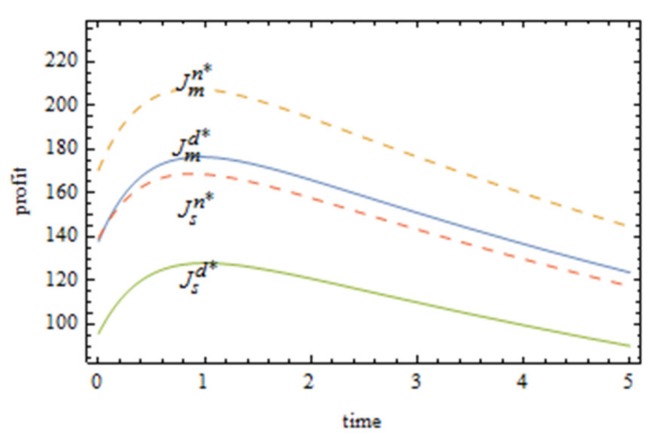
Comparison of profits of manufacturers and suppliers before and after cost sharing.

From Figure 2, the Pareto improvement of the profit of manufacturers and suppliers can be achieved with the cost-sharing contract, and the improvement effect of the profit of suppliers is better than that to the profit of manufacturers. Because manufacturers share the cost of the green innovation efforts of suppliers, the optimal level of the green innovation efforts of suppliers will be improved; the greenness of products will be improved and the market demand will be increased. This will make the profits of both sides reach Pareto improvement. Over time, the profits of both manufacturers and suppliers tend to rise first and then decline, indicating that, in the short term, coordinated green innovation efforts between manufacturers and suppliers can bring considerable economic benefits to both. However, due to the dual externalities of green innovation efforts themselves (spillover effect and external environmental cost), the technical difficulty of green innovation becomes more and more difficult, the input cost keeps increasing, and the profit of manufacturers and suppliers keeps declining in the long term. Therefore, if long-term benefits are required for green innovation efforts in the food supply chain, support from the government and other green groups has to be considered.

**Figure 3:**
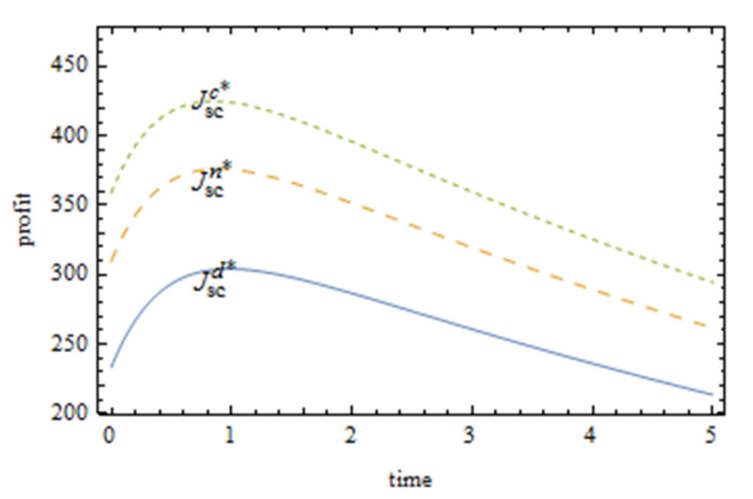
Comparison of food supply chain system profits under three conditions.

It can be seen from Figure 3 that the profit ranking of three supply chain system cases from the best to the worst is as follows: the food supply chain with centralized decision making, the food supply chain with cost sharing, and the food supply chain with no cost sharing. It shows that the cost sharing contract can help the Pareto improvement of the food supply chain system profit, but cannot optimize the Pareto to the best case. From the distance between the three curves in Figure 2, it can be intuitively seen that the cost-sharing contract has an obvious improvement effect on the profit of the food supply chain. As the upper limit of the green innovation efforts of the food supply chain, centralized decision making serves as the reference basis for food supply chain enterprises to carry out collaborative green innovation. With the profit of the food supply chain under centralized decision making as the benchmark, manufacturers can constantly adjust their sharing strategy for suppliers and decide whether to share the cost and proportion with suppliers, so as to maximize the profit of the food supply chain.

**Figure 4:**
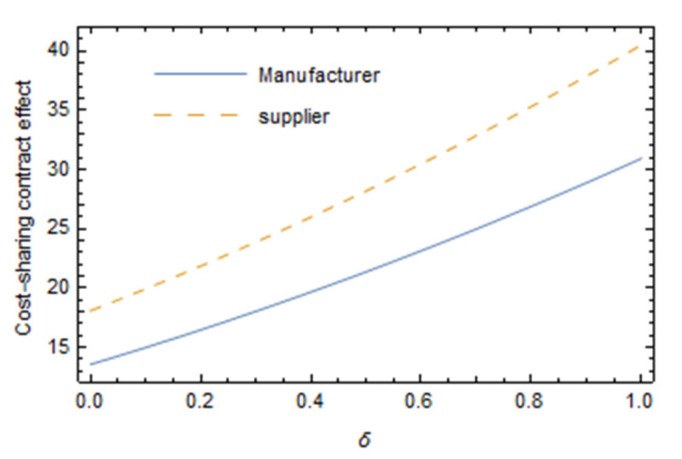
Effect of parameter δ on the Pareto improvement of a cost-sharing contract.

As can be seen from Figure 4, with the increase in the sensitivity of the consumer reference price effect, a cost-sharing contract has a clearer effect on Pareto improvement of manufacturer and supplier profit, and the effect of a cost-sharing contract on supplier improvement is better than that on manufacturer improvement. The clearer the reference price effect of consumers (that is, consumers have higher requirements on the price of green products), the more the consumers will choose to give up buying products if the price is much higher than the “reference price”. Consumers make a purchase decision only when the price of green products is within the acceptable reference range. In this case, collaborative green innovation efforts between manufacturers and suppliers can reduce the cost of green innovation efforts in the food supply chain under the condition of improving the greenness of the same products, so that green products can occupy a certain price advantage in the competition. This can provide a reference for manufacturers to make decisions. Manufacturers can compare the participation of suppliers in green innovation efforts to decide whether to increase subsidies for suppliers and the amount of subsidies to win more market opportunities.

**Figure 5:**
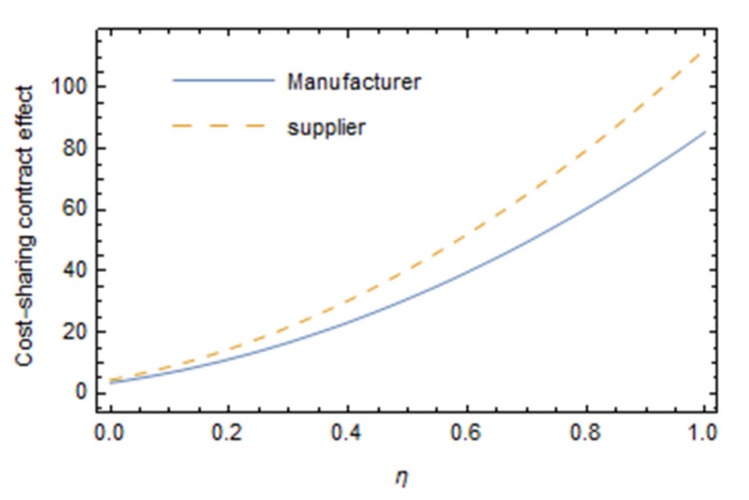
Influence of parameters on the Pareto improvement effect of a cost-sharing contract.

It can be seen from Figure 5 that the Pareto improvement effect of a cost-sharing contract on the profit of manufacturers and suppliers is clearer with the increase in the green preference coefficient of consumers, and the improvement effect of cost-sharing contract on suppliers is better than that on manufacturers. The higher the green preference coefficient of consumers, the more green products they prefer, and the more they are even willing to pay more green premiums for green products. As the market demand increases, the profits of manufacturers and suppliers increase accordingly. Compared with Figure 3, it can be found that the influence of consumers’ green preference on the improvement effect of a cost-sharing contract is greater than that of the reference price effect. With the increase in consumers’ green preference coefficient, the profit difference between manufacturers and suppliers increases rapidly before and after cost sharing.

## 5. Conclusions

In summary, the interactive influences of consumers’ green preference and the reference price effect on decision making in terms of green innovation in the food supply chain are examined empirically in this paper. This is because there is not enough research examining the interactive effects of consumer behavior and companies’ strategic behavior on the results of green innovation. The effects of decentralized decision making and centralized decision making on the level of the green innovation efforts of manufacturers and suppliers in the food supply chain are discussed, and the differences in the decision making results under various scenarios are compared. The main conclusions are as follows.

First, whether decentralized decision making or centralized decision making, consumers’ green preference parameters and reference price effect parameters are positively correlated with the level of green innovation efforts of the main body of the food supply chain. However, in different decision-making scenarios, there are significant differences in the level of green innovation efforts of the food supply chain. In more detail, with the decentralized decision making of cost-free sharing, the level of effort of both is the lowest; with the decentralized decision making of cost sharing, the level of effort of suppliers has been improved, while that of manufacturers remains unchanged; with centralized decision making, the level of effort of both is the highest.

Secondly, in the case of consumers’ green preference and the reference price effect, food manufacturers’ cost subsidies for the green innovation efforts of suppliers will stimulate their green innovation efforts. This phenomenon has rarely been addressed in previous research on green innovation [8]. However, the realization of such subsidies requires certain threshold conditions, which means that only when suppliers’ marginal profit is less than twice that of manufacturers are subsidies useful to stimulate the green innovation efforts of suppliers. 

Thirdly, in the process of promoting green innovation efforts in the food supply chain, there is an interaction between consumers’ green preference and the reference price effect, which further strengthens the incentive role of the green innovation efforts of food supply chain members. This means that consumers’ strong green preference will weaken their reference price effect in terms of decision making when purchasing green products, making consumers more willing to purchase green products and pay more green premiums for products. This will encourage manufacturers to share the green innovation cost of suppliers and promote the efficiency and sustainability of green innovation in the food supply chain.

There are still some limitations in this paper, which need to be further expanded in future research. Firstly, in order to simplify the model, only green innovation efforts carried out by a single supplier and a single manufacturer are considered. However, in practice, large manufacturers are more willing to cooperate with multiple suppliers in order to find the best combination of benefits. Further research can be conducted with consideration of the cooperative green innovation of the food supply chain under the competition of multiple suppliers. Secondly, consumers’ green preferences and the influence of the reference price effect on green innovation decision making in the food supply chain may be an indirect or non-linear process. It is also affected by factors such as government subsidies and market size. The existence and influence of these factors can be further explored in future research.
